# Supplementary figures and images for: Response to resources and parasites depends on health status in extensively grazed sheep
Source: Proc Biol Sci. 2020 Feb 5;287(1920):20192905. doi: 10.1098/rspb.2019.2905 (PMC7031671; doi:10.1098/rspb.2019.2905)

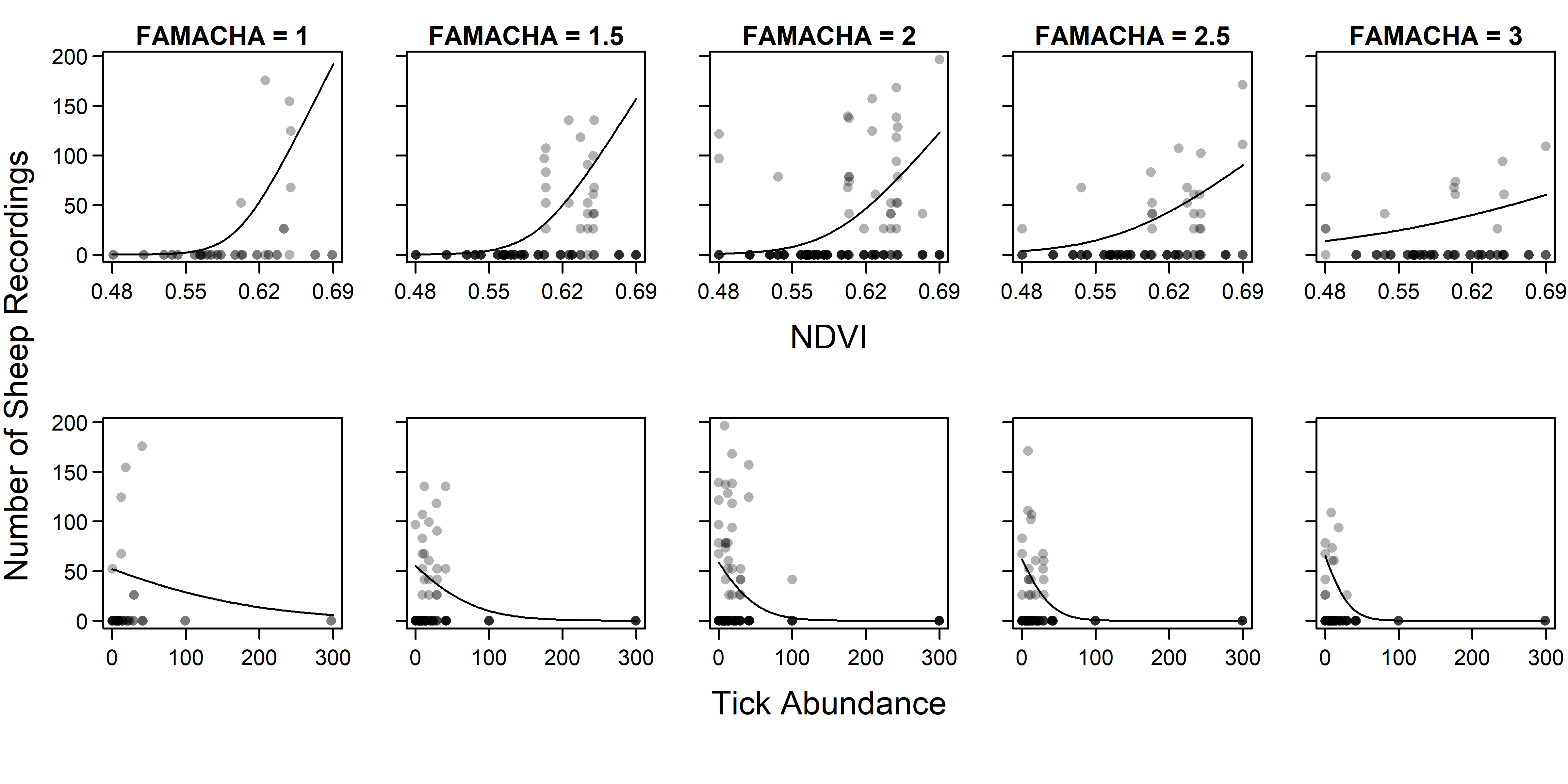

Supplement: Figure S1 [file rspb20192905supp1.tiff]

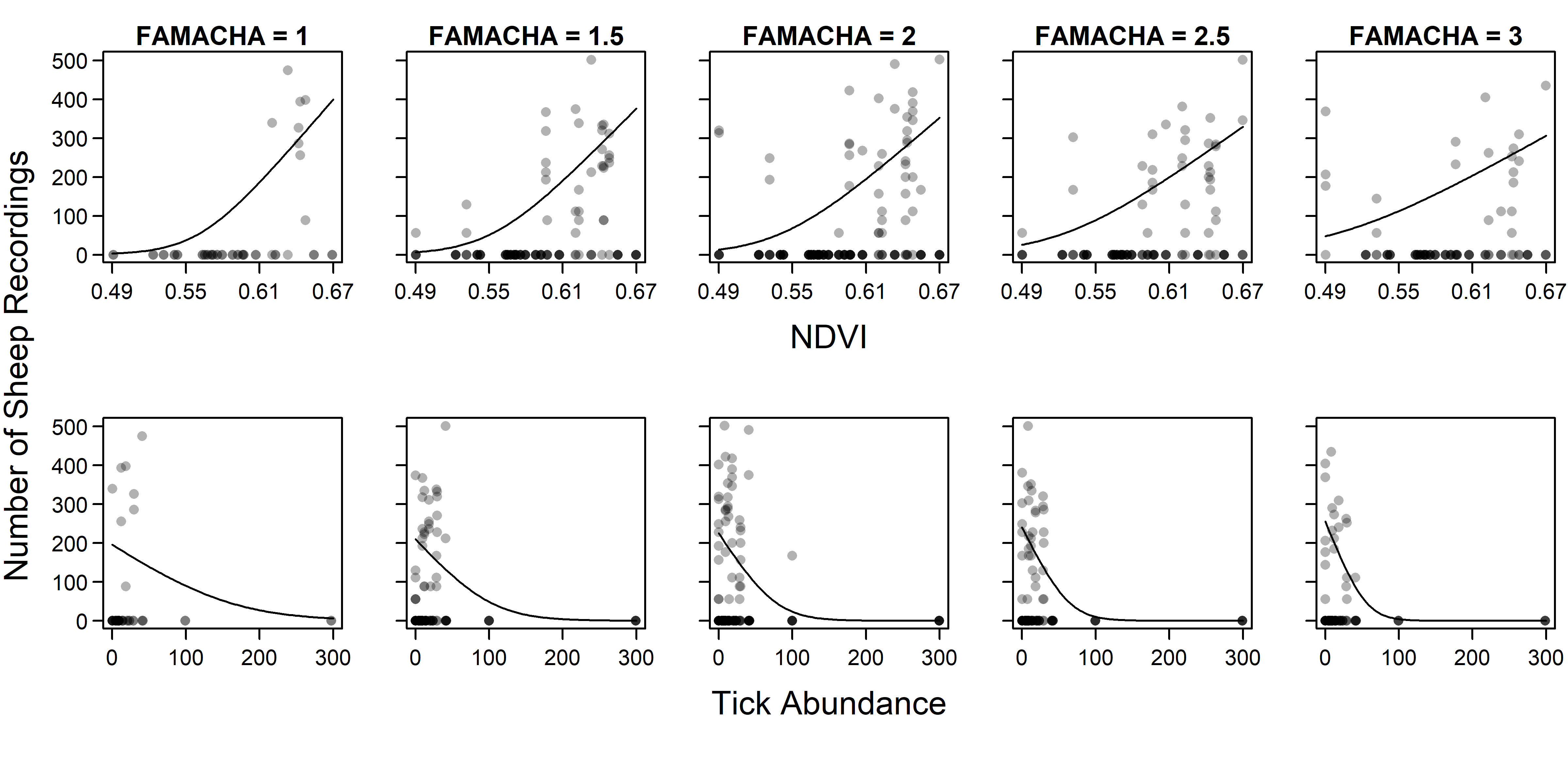

Supplement: Figure S2 [file rspb20192905supp2.tiff]

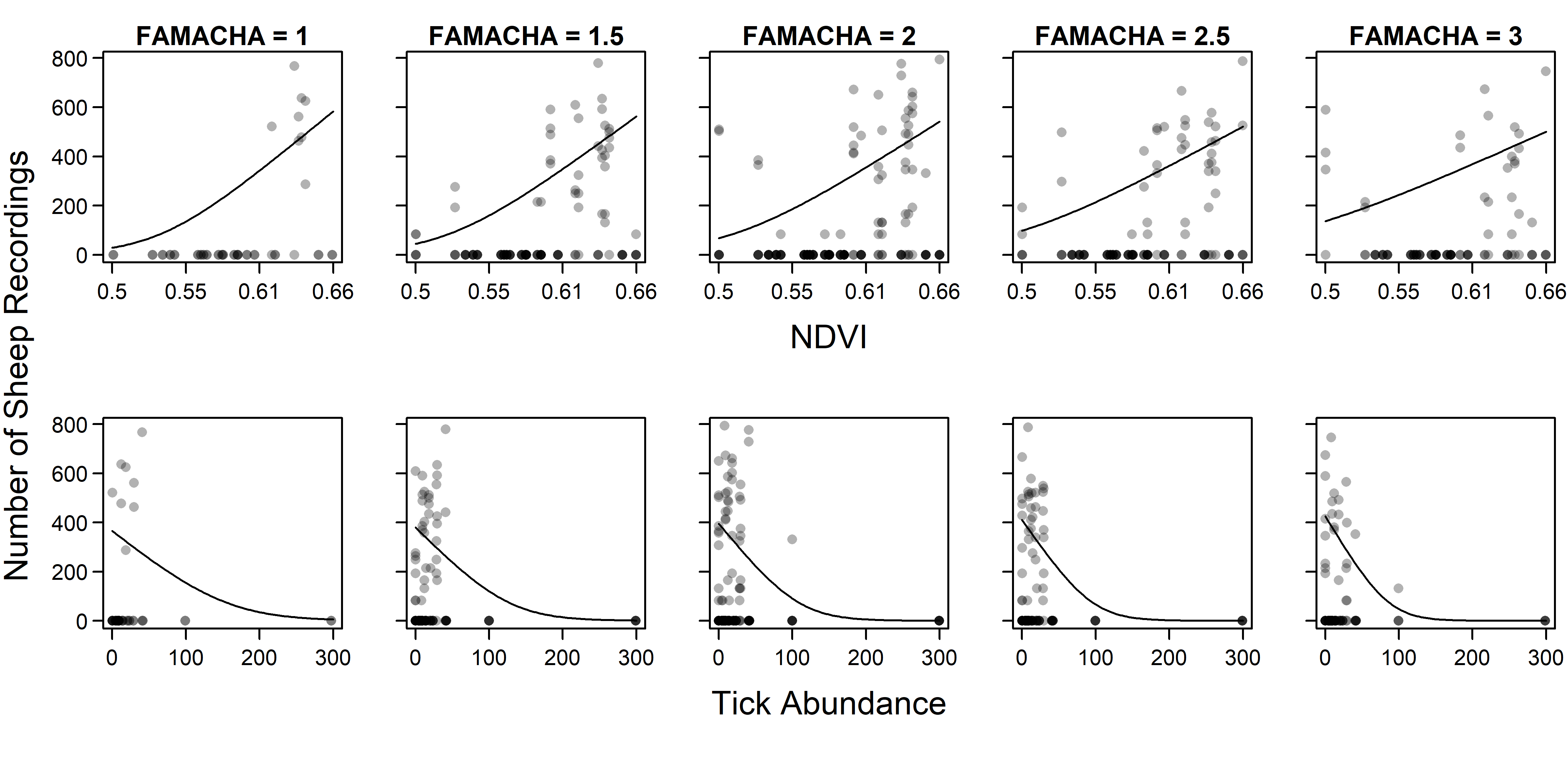

Supplement: Figure S3 [file rspb20192905supp3.tiff]
